# Large seasonal variation of soil respiration in a secondary tropical moist forest in Puerto Rico

**DOI:** 10.1002/ece3.7021

**Published:** 2020-12-10

**Authors:** Omar Gutiérrez del Arroyo, Tana E. Wood

**Affiliations:** ^1^ Department of Biology University of Puerto Rico Río Piedras Puerto Rico; ^2^ USDA Forest Service International Institute of Tropical Forestry Rio Piedras Puerto Rico; ^3^ Department of Environmental Science, Policy, and Management University of California Berkeley California

**Keywords:** litterfall, moisture, nutrients, seasonality, soil respiration, temperature, tropical forests

## Abstract

Tropical forests are the largest contributors to global emissions of carbon dioxide (CO_2_) to the atmosphere via soil respiration (*R*_s_). As such, identifying the main controls on *R*_s_ in tropical forests is essential for accurately projecting the consequences of ongoing and future global environmental changes to the global C cycle. We measured hourly *R*_s_ in a secondary tropical moist forest in Puerto Rico over a 3‐year period to (a) quantify the magnitude of *R*_s_ and (b) identify the role of climatic, substrate, and nutrient controls on the seasonality of *R*_s_. Across 3 years of measurements, mean *R*_s_ was 7.16 ± 0.02 μmol CO_2_ m^‐2^ s^‐1^ (or 2,710 g C m^‐2^ year^‐1^) and showed significant seasonal variation. Despite small month‐to‐month variation in temperature (~4°C), we found significant positive relationships between daily and monthly *R*_s_ with both air and soil temperature, highlighting the importance of temperature as a driver of *R*_s_ even in warm ecosystems, such as tropical forests. We also found a significant parabolic relationship between mean daily volumetric soil moisture and mean daily *R*_s_, with an optimal moisture value of 0.34 m^3^ m^‐3^. Given the relatively consistent climate at this site, the large range in mean monthly *R*_s_ (~7 μmol CO_2_ m^‐2^ s^‐1^) was surprising and suggests that even small changes in climate can have large implications for ecosystem respiration. The strong positive relationship of *R*_s_ with temperature at monthly timescales particularly stands out, as moisture is usually considered a stronger control of *R*_s_ in tropical forests that already experience warm temperatures year‐round. Moreover, our results revealed the strong seasonality of *R*_s_ in tropical moist forests, which given its high magnitude, can represent a significant contribution to the seasonal patterns of atmospheric (CO_2_) globally.

## Graphical abstract

We measured hourly soil respiration in a tropical moist forest in Puerto Rico over a 3‐year period to (a) quantify the magnitude of *R*_s_ and (b) identify the role of climatic, substrate, and nutrient controls on the seasonality of *R*_s_. Across 3 years of measurements, mean *R*_s_ was 7.08 ± 0.02 μmol CO2 m‐2 s‐1 (2,728 g C m^‐2^ year^‐1^) and showed significant seasonal variation at daily and monthly timescales. Despite small month‐to‐month variation in temperature (~4 C), seasonal patterns of daily and monthly *R*_s_ were strongly correlated with air temperature (*R* ^2^ ranged from 0.49 to 0.70 depending on the timescale), highlighting the importance of temperature as a driver of *R*_s_ even in warm ecosystems, such as tropical forests.

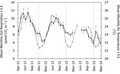

## 1 INTRODUCTION

Tropical moist and wet forests are the largest contributors to global emissions of carbon dioxide (CO_2_) to the atmosphere via soil respiration (*R*_s_), as warm temperatures and abundant rainfall allow for high rates of primary production and decomposition year‐round (Bond‐Lamberty & Thomson, 2010; Raich & Schlesinger, 1992; Rubio & Detto, 2017; Schlesinger & Andrews, 2000). Since *R*_s_ represents a significant fraction of total ecosystem respiration, small changes in this large flux could shift the carbon (C) balance of tropical forests affecting the C sequestration potential of these ecosystems (Chambers et al., 2004; Saleska et al., 2003). Given the disproportionate role tropical forests play in the global C cycle, a potential decrease in C uptake might further accelerate the buildup of atmospheric (CO_2_) (Clark, 2004). Therefore, constraining the response of *R*_s_ to natural variability in climate in tropical forests is essential for accurately projecting the consequences of ongoing and future global environmental changes on the global C cycle.

In forest ecosystems, *R*_s_ is dominated by microbial (i.e., heterotrophic), and root‐rhizosphere (i.e., autotrophic) CO_2_ production (Hanson et al., 2000; Ryan & Law, 2005). These processes are driven by a diverse set of organisms, including plants, fungi, and bacteria, which inherently have differential sensitivities to environmental changes (Cattânio et al., 2002; Manzoni et al., 2011; Metcalfe et al., 2007; Subke et al., 2006). Moreover, these organisms inhabit a structurally complex matrix—the soil—where resource availability is highly heterogeneous in part due to microclimate variability at the pore scale. This inherent complexity of soils, as well as the range of processes occurring within it, often hinders research efforts seeking to identifying the main controls of *R*_s_, especially in tropical forest ecosystems, which are considered hot spots of biogeochemical heterogeneity (Townsend et al., 2008). An improved understanding of the seasonal drivers of *R*_s_ in tropical forests would provide valuable insights as to what mechanisms are driving the temporal patterns of *R*_s_, as well as their respective sensitivities to a changing climate.

Observational studies have highlighted the importance of precipitation regimes and soil moisture availability in regulating the seasonality of *R*_s_, especially in sites with a narrow intraannual range of seasonal temperatures. These studies have shown that highest rates of *R*_s_ usually occur during the wet season, at intermediate levels of soil moisture, when conditions are optimal for biological activity in the soil (Chambers et al., 2004; Rubio & Detto, 2017; Schwendenmann et al., 2003; Sotta et al., 2006; Wood et al., 2013). During periods of excess soil moisture, *R*_s_ often declines due to limited soil–atmosphere gas exchange, which inhibits aerobic decomposition as oxygen is depleted (Liptzin et al., 2010; Silver et al., 1999; Sotta et al., 2004). However, under low redox conditions, anaerobic pathways (i.e., Fe(II) reduction) can contribute a significant fraction of *R*_s_, especially in tropical wet forests that often experience fluctuating soil redox dynamics (Dubinsky et al., 2010; Hall et al., 2015). At the other extreme, low soil moisture tends to decrease *R*_s_, mainly due to lower rates of fine root growth and reduced microbial decomposition under suboptimal conditions of water and nutrient availability (Cattânio et al., 2002; Manzoni et al., 2011; Sotta et al., 2007; Wood & Silver, 2012). Alternatively, in aseasonal tropical wet forests where there is often an excess in water availability, improved aeration of the soil matrix and more concentrated pulses of dissolved organic matter during dry periods may actually stimulate *R*_s_, as well as rates of nitrogen (N) and phosphorus (P) cycling (Cleveland et al., 2010; Wieder et al., 2011).

In addition to soil moisture, multiple studies have also demonstrated the important role of temperature as a driver of *R*_s_, even in warm tropical forests with low climate seasonality (Raich, 2017; Schwendenmann & Veldkamp, 2006; Sotta et al., 2004; Sotta et al., 2006; Valentini et al., 2008; Wood et al., 2013). Both short‐term laboratory incubations and field studies suggest that temperature exerts a strong control on *R*_s_ by increasing rates of microbial decomposition, especially when moisture, nutrients, and C are not limiting (Holland et al., 2000; Wood et al., 2013). However, apart from the direct kinetic effect of warming on enzyme activity (Lloyd & Taylor, 1994), temperature can also be an indirect control on *R*_s_ by affecting rates of primary productivity, patterns of C allocation, and soil nutrient availability (Medina & Zelwer, 1972; Vargas et al., 2010; Wood et al., 2012) ). For example, slight seasonal changes in climate (i.e., temperature, precipitation), which are known to affect microbial activity, may coincide with changes in plant phenology (i.e., root growth or litterfall pulses), effectively confounding the primary controls on the seasonality of *R*_s_ (Curiel Yuste et al., 2004; Vose & Ryan, 2002). Thus, it is critical to consider the potentially confounding effects of seasonality of C inputs (i.e., litter or root exudates) or other climatic variables (i.e., moisture, light) when studying the temperature sensitivity of *R*_s_.

Motivated by the global relevance of *R*_s_ in tropical forests, as well as the complexity of its mechanistic drivers, research efforts over the last several decades have significantly advanced our understanding of the physical and biological factors that regulate the temporal variation of *R*_s_ (Meir, Wood, et al., 2015; Rubio & Detto, 2017; Vargas et al., 2010). However, most studies that have focused on the seasonal variation of *R*_s_ in tropical forests rely on data sets with low temporal resolution, mostly consisting of monthly or biweekly sampling over a 1‐year period. Moreover, most studies are based in undisturbed forest sites, with only a few measuring *R*_s_ in secondary forests, which make up a large (and increasing) percentage of forests in the tropics (Asner et al., 2009; Gómez‐Pompa & Vázquez‐Yanes, 1974; Lugo, 2009). To bridge this gap, automated *R*_s_ systems provide a formidable tool for collecting long‐term (i.e., multi‐year), continuous data sets with high temporal resolution (i.e., hourly) that allow us to finely dissect the seasonal variation of *R*_s_ across changing ecosystems (Savage et al., 2009; Vargas & Allen, 2008; Vargas, Detto, et al., 2010). In this context, we collected hourly *R*_s_ measurements over a 3‐year period to (a) quantify the magnitude of *R*_s_ and (b) identify the role of climatic, substrate, and nutrient controls on the seasonality of *R*_s_ in a tropical moist forest in Puerto Rico. Controls on soil respiration at diel timescales are explored in a separate publication (Gutiérrez del Arroyo & Wood, 2020).

## 2 MATERIALS AND METHODS

### 2.1 Study site

This study was conducted in a private nature reserve (*El Tallonal*), consisting of 114 ha of forested limestone hills in northwestern Puerto Rico at ~ 100 m a.s.l. (18º24’27” N 66º43’53” W). The site is classified as a subtropical moist forest (Ewel & Whitmore, 1973) according to the Holdridge life zone system (Holdridge, 1967). Mean annual temperature is 23°C and ranges from ~25°C in July and August to ~21°C in January (annual range of ~4°C among months). Mean annual precipitation from 1999 to 2013 was 2,016 ± 126 mm, with a short dry season (<100 mm per month) usually lasting from December to March, which coincides with the coolest months of the year (i.e., cool and dry season; personal communication Mr. Abel Vale). Soils are slightly acidic (pH = 5–6), clay‐rich oxisols derived from the weathering of volcaniclastic parent material from upland mountains (Martínez et al., 2008). At 0–10 cm soil depth, soil C and N content is 4.4% and 0.4%, respectively, and Olsen‐extractable P is 16.9 μg/g (Gutiérrez del Arroyo, 2014). Sand, clay, and silt particles made up 39%, 35%, and 26% of the soil, respectively, classifying the soil texture as a clay loam (Gutiérrez del Arroyo, 2014).

The study site is a mature, secondary forest (~60 years at the time of study) which has regenerated from prior agricultural use and cattle grazing (Fonseca da Silva, 2015). Vegetation is dominated by *Castilla elastica* Sessé (Moraceae), an introduced tree species in Puerto Rico, alongside a mix of several other native tree species, making this a novel secondary forest (Hobbs et al., 2006; Lugo & Helmer, 2004). The dominance of the naturalized tree *C. elastica* in this forest is demonstrated by its importance value index of 37% (Fonseca da Silva, 2014).

### 2.2 Experimental design

We measured hourly *R*_s_ using six automated chambers (permanently installed) connected to an infrared gas analyzer throughout a 3‐year period from March 2011 to March 2014 (Li‐Cor LI‐8100/8150 Multiplexer; Li‐Cor Biosciences). One month prior to starting our measurements, the six‐chamber collars (20 cm diameter) were permanently inserted 2–4 cm into the soil at a mean distance of ~ 5 m in a semicircular shape within a ~ 600‐m^2^ forested area. We were careful to maintain the litter layer in place during chamber installation and made sure the affected area continued receiving normal inputs of litterfall throughout our study period (i.e., chambers remained open while not measuring). Although relatively uncommon, seedlings that sprouted within the chambers were clipped at the base of their stem to prevent the confounding effects of foliar respiration. Hourly soil respiration values for each chamber were calculated by the Li‐Cor FluxPro software based on the exponential increase in CO_2_ concentrations during a 1.5‐min period, after a 45 s prepurge period between measurements. From April 2013 to June 2013, soil respiration was not measured due to necessary maintenance of the LI‐8100 infrared gas analyzer (light sensor replacement and recalibration).

On November 2012, we installed soil temperature sensors (TMC‐50HD) at 5 cm depth and volumetric soil moisture sensors (S‐SMD‐M005) at 0–10 cm depth, both within 0.5 m of each chamber, and measuring at hourly intervals. On January 2013, we equipped a 25‐meter scaffolding tower in the forest with sensors for measuring hourly values of air temperature, relative humidity, and photosynthetically active radiation at the forest canopy (PAR; S‐THB‐M002; S‐LIA‐M003; Onset Computer Corporation). A meteorological station within the private natural reserve (<1 km away) provided monthly precipitation from two independent methods: a manual gauge that provides total monthly precipitation and an automated precipitation gauge that measured at 15‐min intervals (Onset Computer Corporation). Additionally, air temperature, relative humidity, soil moisture, and PAR were also measured at 15‐min intervals using sensors installed at the meteorological tower (S‐THB‐M002; S‐SMD‐M005; S‐LIA‐M003; Onset Computer Corporation). All data collected at subhourly frequencies were aggregated to hourly sampling intervals to maintain consistency across data sets. Considering the data set from the meteorological station covers a longer time period than the measurements conducted in the forest (3 vs. 1 years), we used air temperature data collected at the meteorological station for all analyses. Furthermore, we used the empirical relationship between mean monthly air temperature and soil temperature to estimate the seasonal pattern of soil temperature across the 3‐year period.

From March 2013 to February 2014 (1‐year period), we collected litterfall every 2 weeks using six 0.25 m^2^ baskets (constructed with PVC tubing and fiberglass mesh), each placed at least 3 m away from each chamber. Litterfall was dried at 65°C to a constant weight, and then leaves were separated from all other litterfall to measure their dry weights. Concurrent with measurements of litterfall, we measured the flux of soil macro‐ and micronutrients (including N, P, K, Ca, Mg, Fe, Al) using three pairs of PRS™‐probes (Western Ag Innovations Inc., Saskatoon, SK, Canada). These were buried at 5 cm depth for 4‐week intervals within 1.5 m of each chamber. Nutrients extracted from the three pairs of PRS™‐probes (paired probes target cations and anions, separately) associated with each chamber were pooled to give one value per chamber for each 4‐week sampling interval.

### 2.3 Data analysis

Regression analysis was used to analyze the relationships between measured variables (i.e., climate, litterfall, soil nutrients) at daily and monthly timescales (Sigma Plot, Systat Software, Inc., 2015). Significant differences in *R*_s_ among months were determined using a generalized linear mixed model (Proc Mixed Repeated in SAS; SAS for Windows V8.0, 2002, SAS Institute), with chamber as the random factor. We tested the soil respiration data for homogeneity of variance and log transformed when assumptions were not met.

Occasionally, *R*_s_ data from individual chambers were affected by mechanical problems, fallen branches, or land snails common at the study site, all of which impeded proper closure of the chambers. These values were identified using field notes and visual examination of the data and were excluded from further analyses. Following heavy precipitation events, measured *R*_s_ values were often zero or even slightly negative, likely due to flooding of the soil collars. Because soil CO_2_ consumption is unlikely to occur in this system, we converted negative values of *R*_s_ to zero (<0.1% of data set).

While the goal of this study was to evaluate seasonal variation in soil respiration, these data also allow for analyses at diel timescales. We explore patterns of soil respiration at diel timescales in detail in a separate publication (Gutiérrez del Arroyo & Wood, 2020).

## 3 RESULTS

### 3.1 Climate

**Figure 1:**
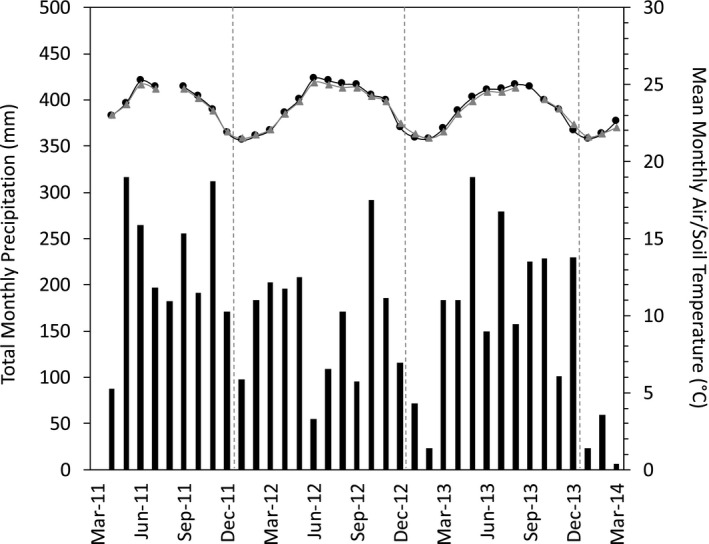
Seasonal patterns of total monthly precipitation (gray bars) and mean monthly air (black circles/line) and soil temperature (gray triangles/line) from April 2011 to March 2014. Missing values for soil temperature were estimated using the equation from the significant positive regression with measured mean monthly air temperature (*R* ^2^ = .94,*p* < .0001). Vertical dashed gray lines indicate separate years (2011–2014)

Mean air temperature throughout the study period was 23.5°C, with monthly means ranging from 21.5 to 25.3°C, in January and June, respectively (Figure 1). Interannual variation in the seasonal pattern of air temperature was minimal, with warming occurring at a faster rate from January to June, compared to the corresponding cooling from June to December (Figure 1). Mean soil temperature at 5 cm was 23.2°C, with monthly means following a similar seasonal pattern as air temperature, showing a slightly buffered range among months (~3.5°C).

Mean annual precipitation from 2011 to 2013 was 2,119 ± 113 mm (the 9‐year mean was 2,016 mm). Although total annual precipitation remained relatively stable throughout our study period, total monthly precipitation was highly variable, ranging from 23 to 316 mm (Figure 1). Overall, there were two peaks of rainfall occurring in May and October, while the driest conditions occurred from December to March (Figure 1). However, the timing of high and low rainfall varied across years. For example, total precipitation during June 2011 was >250 mm, but only 55 mm in June 2012 (Figure 1). Additionally, during 2012, the typical seasonal pattern of precipitation was modified, with above‐average total monthly precipitation during the dry season and low precipitation (~100 mm/month) during the summer (June to September; Figure 1).

Volumetric soil moisture measured at the meteorological station (<1 km away) and at the forest, showed marked temporal fluctuations in response to precipitation events. Maximum volumetric soil moisture was higher at the forest than at the meteorological station, but there was a similar response to extended dry periods at both sites. Daily mean volumetric soil moisture tended to be lower at the meteorological station compared to the forest station, with values ranging from 0.12 to 0.37 m^3^ m^‐3^ (3‐year period) and 0.24 to 0.45 m^3^ m^‐3^ (1‐year period) at the respective sites. The temporal dynamics of volumetric soil moisture were similar at both sites, where extended dry periods resulted in a constant reduction in moisture until the next precipitation event. During the dry season (December–March), extended periods with little or no precipitation were more frequent and prolonged, resulting in the lowest measured values of volumetric soil moisture at both sites (~0.15 and 0.25 m^3^ m^‐3^ at meteorological station and forest, respectively).

Mean daily relative humidity at the meteorological station ranged from 83% to 100%, and 85 to 100% at the forest station (i.e., forest canopy). At both sites, there was a seasonal trend in mean daily relative humidity, with humidity being more variable during the spring and summer (April–September) when compared to the constantly high humidity observed during the cooler months (November–March). Mean daily PAR measured at the forest canopy, despite being highly variable due to cloudiness, also showed a seasonal trend with higher values occurring during August and the lowest in December.

### 3.2 Nutrients

We observed strong seasonal variation in the flux of macro and micronutrients in surface soils, as measured by the PRS‐Probes. For example, total inorganic *N* (ammonium plus nitrate) ranged from 12 μg 10‐cm^‐2^ 28‐days^‐1^ at the beginning of the dry season (December 2013) to 50 μg 10‐cm^‐2^ 28‐days^‐1^ at the end of the dry season (March 2014). This temporal variation was largely driven by changes in nitrate availability, which dominated the total inorganic N pool throughout the year (~80% of total N). Conversely, there was no evident seasonal trend in the flux of P (mean: 2.6 ± 0.25 μg 10‐cm^‐2^ 28‐days^‐1^), which showed high spatial variation among chambers. There was a marked seasonality in the flux of K (annual range: 9 to 73 μg 10‐cm^‐2^ 28‐days^‐1^), with values showing an increase during the dry season, relative to the rest of the year. An opposite trend was measured for iron (Fe) and manganese (Mn), with the flux of both nutrients peaking during the wetter season (June and November 2013). Similarly, the flux of Al also showed a seasonal trend, peaking in August and gradually decreasing toward the dry season. Calcium and magnesium followed a weak seasonal trend, with the lowest values for both nutrients measured during the dry season (March 2014).

### 3.3 Litterfall

**Figure 2:**
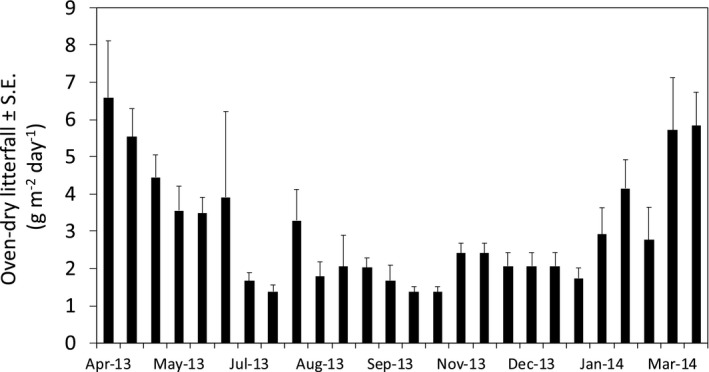
Seasonal pattern of biweekly litterfall with standard errors (*n* = 6) across a 1‐year period from April 2013 to March 2014

Total annual litterfall was 11 ± 1.3 Mg/ha year^‐1^ (*n* = 6), with leaf‐fall making up nearly 90% of total annual litterfall. Litterfall showed a marked seasonal pattern, with a large peak in March and April, coinciding with the period of leaf drop of the dominant tree species, *C. elastica* (Figure 2). Across the study period, biweekly rates of litterfall ranged from 19 g/m^2^ in October 2013, to 92 g/m^2^ in April 2013.

### 3.4 Soil respiration

**Figure 3:**
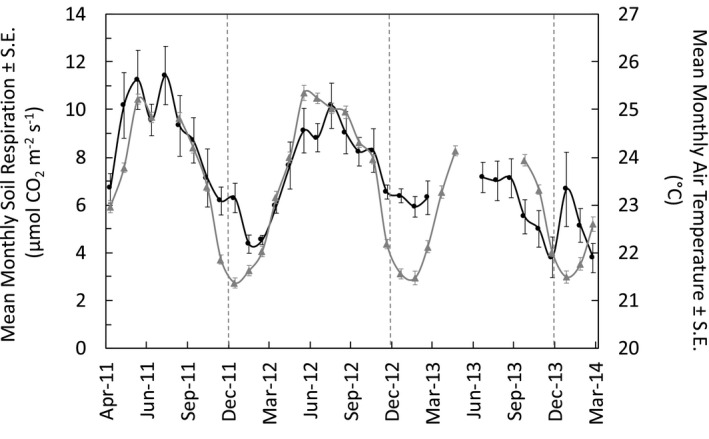
Seasonal patterns of mean monthly air temperature (gray line/triangles) and soil respiration (black line/circles) with standard errors across a 3‐year period from April 2011 to March 2014 (*n* = 5–6)

**Figure 4:**
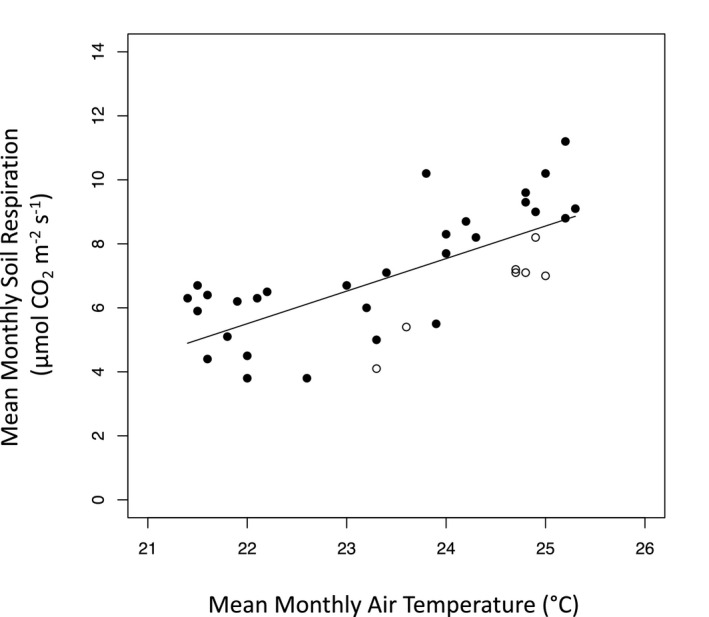
Significant positive linear regression between mean monthly air temperature and soil respiration (*R* ^2^ = .48,*p* < .0001). Black points indicate measured values for mean monthly air temperature, while open points indicate estimated values for mean monthly air temperature calculated with the equation from the significant positive regression with measured mean monthly soil temperature (*R* ^2^ = .94,*p* < .0001)

Across 3 years of measurements, mean *R*_s_ was 7.16 ± 0.02 μmol CO_2_ m^‐2^ s^‐1^ (or 2,710 g C m^‐2^ year^‐1^) and showed significant seasonal variation at daily and monthly timescales (*F*(11, 171) = 6.83, *p* < .0001; Figure 3). Daily and monthly mean *R*_s_ were 7.17 ± 0.07 and 7.12 ± 0.34 μmol CO_2_ m^‐2^ s^‐1^, respectively. Daily mean *R*_s_ ranged from 1.8 to 14.2 μmol CO_2_ m^‐2^ s^‐1^, while monthly mean *R*_s_ had a narrower range from 3.6 to 11.4 μmol CO_2_ m^‐2^ s^‐1^. We found a significant positive linear regression between *R*_s_ and air temperature, which explained nearly half the seasonal variation of daily *R*_s_ (Figure S1; *R* ^2^ = .48, *p* < .0001, *DailySR* = 1.1 * *DailyAT* – 18.0) and monthly *R*_s_ (Figure 4; *R* ^2^ = .48, *p* < .0001, *MonthlySR* = 1.0 * *MonthlyAT* – 16.9). Mean monthly *R*_s_ also showed a significant positive linear regression with mean monthly soil temperature (Figure S2; *R* ^2^ = .44, *p* < .0001, *MonthlySR* = 1.1 * *MonthlyST* – 20.0). However, although the positive linear regression was still significant at daily timescales, the relationship was much weaker, only explaining around 10% of the observed variation in daily *R*_s_ (Figure S3; *R* ^2^ = .12, *p* < .0001, *DailySR* = 0.5 * *DailyST* – 4.44).

**Figure 5:**
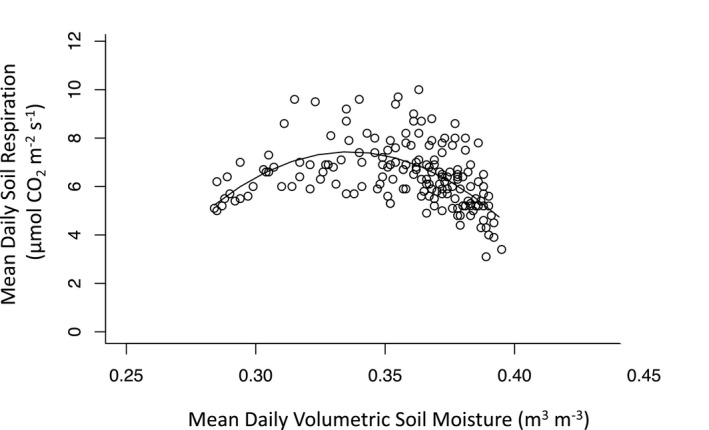
Significant parabolic relationship between mean daily volumetric soil moisture and soil respiration (all modeled parameters for the parabolic equation were significant at*p* < .001;*DailySR*= −806.31 * (*DailyVSM*^2) + (542.20 *DailyVSM) – 83.72)

Precipitation events were also important in determining day‐to‐day variation of *R*_s_, leading to a transient reduction in *R*_s_. We found a significant parabolic relationship between mean daily volumetric soil moisture and mean daily *R*_s_, with an optimal moisture value of ~ 0.34 m^3^ m^‐3^ (Figure 5; all parameters for the parabolic equation were significant at *p* < .001, *DailySR* = −806.31 * (*DailyVSM*^2) + (542.20 * *DailyVSM*) – 83.72). Although we did not find any seasonal relationship between *R*_s_ and key nutrients such as N, P, or K, the availability of Al in the soil was a surprisingly strong predictor of the temporal variation in *R*_s_, showing a significant positive correlation at seasonal timescales (Figure S3; *R* ^2^ = .43, *p* < .05, *MonthlySR* = 0.13 * *MonthlyAl* + 1.20). Unfortunately, equipment malfunction impeded *R*_s_ measurements from being conducted in early 2013, and thus, there was insufficient overlap to explore the relationship between the seasonality of *R*_s_ and litterfall.

## 4 DISCUSSION

Throughout the 3‐year period of our study, we found that *R*_s_ in this secondary tropical moist forest was of high magnitude relative to other tropical forests, especially during periods of peak temperatures and rainfall (Rubio & Detto, 2017). Mean annual *R*_s_ at our site (2,710 g C m^2^ year^‐1^) was more than double the annual mean of *R*_s_ across a range of tropical moist forests (Raich & Schlesinger, 1992) and tropical ecosystems (Bond‐Lamberty & Thomson, 2010). However, estimates of annual *R*_s_ derived from periodic measurements conducted in tropical wet forest in northeastern Puerto Rico are comparable to our site, ranging from 1,250 to 3,700 g C m^‐2^ (Harris, 2006; Raich & Schlesinger, 1992; Wood et al., 2013). Our estimate for mean annual *R*_s_ also fits within reported values for various tropical moist forest sites in Brazil, which ranged from 2,400 to 2,887 g C m^‐2^ year^‐1^ (Sotta et al., 2004; Trumbore et al., 1995; Valentini et al., 2008) but was lower than the value reported for a tropical dry forest in the Yucatan Peninsula following the pass of hurricane Wilma (Vargas & Allen, 2008).

The high *R*_s_ values observed at our site could be reflective of the tropical moist and wet forest biomes, which are often cited as having especially high soil respiration rates (Bond‐Lamberty & Thomson, 2010; Raich & Schlesinger, 1992; Rubio & Detto, 2017). However, that this site is also a secondary forest could further contribute to high soil respiration rates. Early‐to‐mid successional tropical forests are typically characterized as having fast rates of C cycling (Brown & Lugo, 1990; Fonseca da Silva, 2015; Mascaro et al., 2012). For example, high rates of litterfall measured in this forest (~11 Mg/ha year^‐1^), also characteristic of secondary tropical forests, provide a major source of substrate for decomposers, as well as valuable nutrients to sustain biological activity (this study; Fonseca da Silva, 2015; Lugo et al., 2004; Mascaro et al., 2012; Sayer & Tanner, 2010; Vitousek, 1984). Although there was no significant correlation between the seasonal patterns of litterfall and *R*_s_, it is possible that the pulse of litterfall observed at the end of the dry season serves as a major source of C and nutrients (e.g., Fonseca da Silva, 2015), which could partially explain the elevated *R*_s_ during the summer months, when climatic conditions are optimal for the breakdown of accumulated litterfall (Lodge et al., 1994).

Despite small month‐to‐month variation in temperature (<4°C), seasonal patterns of daily and monthly *R*_s_ were strongly correlated with air temperature, pointing to the importance of temperature as a driver of *R*_s_ even in warm tropical forest ecosystems (Nottingham et al., 2020; Schwendenmann & Veldkamp, 2006; Schwendenmann et al., 2003; Sotta et al., 2004; Sotta et al., 2006; Wood et al., 2013). The significant positive linear relationship between *R*_s_ and air temperature in this forest, suggests that soil CO_2_ emissions in tropical forests may increase with projected warming as long as moisture and substrate are not limiting (Nottingham et al., 2020; Stocker et al., 2013; Townsend et al., 1992). The observed relationship between temperature and *R*_s_ may also be related to seasonal variation in rates of primary productivity, which can affect belowground C investment and *R*_s_ (Giardina & Ryan, 2000). Regardless, observed patterns suggest that temperature plays an important role in regulating the temporal variation of *R*_s_ in this forest. Whether the effects of temperature are direct (i.e., increasing rates of heterotrophic respiration) or indirect (i.e., increasing primary productivity) should be further explored using methods that can partition sources of *R*_s_ (e.g., Nottingham et al., 2020).

Soil moisture was also a significant control on daily and seasonal patterns of *R*_s_ in this forest. Specifically, we observed a significant parabolic relationship between *R*_s_ and soil moisture at both timescales, highlighting the importance of water availability as a regulator of biological activity in the soil, as well as soil–atmosphere gas exchange. Similar to observations in other tropical moist and wet forests, the highest rates of *R*_s_ occurred during periods of intermediate soil moisture (Chambers et al., 2004; Fernández‐Bou et al., 2020; Nottingham et al., 2020; Rubio & Detto, 2017; Schwendenmann et al., 2003; Sotta et al., 2004; Wood et al., 2013). At both daily and monthly timescales, the optimal soil moisture for *R*_s_ was ~ 0.350 m^3^ m^‐3^, which matches the threshold values determined at other tropical forests with similarly textured soils (Meir et al., 2015; Sotta et al., 2004; Wood et al., 2013). Additionally, large precipitation events resulted in sharp increases in soil moisture, which likely led to reduced soil–atmosphere gas exchange, resulting in a sudden drop in *R*_s_. In fact, daily mean *R*_s_ values lower than 4 μmol CO_2_ m^‐2^ s^‐1^ were only observed when soil moisture was higher than 0.370 m^3^ m^‐3^, during periods when soil–atmosphere gas exchange was most likely limited. This negative response of *R*_s_ to large precipitation events has also been documented in other tropical forests, and may be caused solely by the physical diffusion barrier created by high levels of soil moisture and/or the microbial response to reduced soil oxygen concentrations (Fernández‐Bou et al., 2020; Schwendenmann et al., 2003; Silver et al., 1999; Sotta et al., 2004; Wood et al., 2013). Further study is needed to ascertain which of the various possible controls dominate the observed response.

The climatic regime in northern Puerto Rico (and most of the Caribbean region) is characterized by a cool and dry season lasting from December to March, which results in the coupling of two key controls of *R*_s_: temperature and moisture (Angeles et al., 2010). This cool and dry period coincided with the lowest monthly means of *R*_s_ across the 3 years of measurements, thus pointing to both temperature and moisture as potentially limiting to soil respiration. While we did expect lower respiration rates to occur when temperature and moisture were both at their lowest, the large range in mean monthly *R*_s_ (~7 μmol CO2 m^‐2^ s^‐1^) was surprising given the small climatic month‐to‐month variation. This large variation in *R*_s_ at seasonal timescales may have significant implications for the ecosystem C balance. Aside from research focused on seasonally dry tropical forests, there are few data quantifying the seasonality of ecosystem C fluxes in tropical moist and wet forests, despite their disproportionate contributions to the global C cycle through high rates of both primary productivity and respiration (Doughty et al., 2015). Given the major role tropical forests play within the global C cycle, constraining the climate sensitivity of the different components of *R*_s_ will be critical for projecting the response of this large C flux to future climate regimes.

Apart from the climatic drivers, we expected seasonal variability in soil nutrient flux affect *R*_s_. However, we found no significant correlations between monthly *R*_s_ and the primary nutrients thought to limit productivity and/or decomposition in tropical forests (Cuevas & Medina, 1988; LeBauer & Treseder, 2008; Vitousek et al., 2010). The lack of a relationship between *R*_s_ and soil N or P flux may be due either to the form of nutrients measured (resin‐extractable inorganic forms) or to the timescale considered (monthly intervals). Instead, we found a significant positive correlation between soil Al and *R*_s_, which was surprising because Al is a nonessential element that is generally considered an indicator of soil toxicity for plants. Soils in this forest are characterized by having high Al and Fe content, as well as a slightly acidic pH, suggesting that possible variations in soil redox conditions throughout the year may be driving the seasonal changes in Al solubility (Martínez et al., 2008). Moreover, the strong positive exponential correlation between soil Al and monthly precipitation supports our hypothesis that soil redox changes may be an important regulator of the seasonality of *R*_s_ in this tropical moist forest (Hall et al., 2012; Silver et al., 1999). Further research exploring potential controls of redox dynamics in this system is needed.

Overall, the large, 3‐fold intraannual variation of *R*_s_ challenges the paradigm of aseasonality in tropical moist and wet forests. Although it is true that conditions are generally favorable for biological activity year‐round, seasonal fluctuations in incoming solar radiation, temperature and precipitation, can induce significant changes in the rate of ecosystem processes that contribute to *R*_s_, such as litterfall or decomposition. Considering the high magnitude of *R*_s_ in these ecosystems, which exceeds the cumulative annual *R*_s_ of higher latitude ecosystems (Bond‐Lamberty & Thomson, 2010; Fenn et al., 2010; Giasson et al., 2013; Subke et al., 2003), as well as the significant seasonality observed, it is likely that tropical moist and wet forests play a role in regulating the annual fluctuations of atmospheric (CO_2_). Therefore, understanding how a changing climate (i.e., warming and precipitation regime shifts) will affect the seasonality of *R*_s_ across tropical forests will be key for accurately projecting future atmospheric (CO_2_).

## CONFLICT OF INTERESTS

The authors have no competing interests to declare.

## AUTHOR CONTRIBUTION

Omar Gutierrez del Arroyo: Conceptualization (equal); Data curation (equal); Formal analysis (equal); Funding acquisition (equal); Investigation (equal); Methodology (equal); Project administration (equal); Resources (equal); Software (equal); Supervision (equal); Validation (equal); Visualization (equal); Writing‐original draft (equal); Writing‐review & editing (equal). Tana Wood: Conceptualization (equal); Data curation (equal); Formal analysis (equal); Funding acquisition (equal); Investigation (equal); Methodology (equal); Project administration (equal); Resources (equal); Software (equal); Supervision (equal); Validation (equal); Visualization (equal); Writing‐original draft (equal); Writing‐review & editing (equal).

## AUTHOR CONTRIBUTIONS

Both authors contributed equally to this manuscript.

## Supporting information

Fig S1

Fig S2

Fig S3

## Data Availability

Our complete data sets are available in USDA Forest Service Research Data Archive (https://doi.org/10.2737/RDS‐2020‐0011).
