# Supplementary figures and images for: Large seasonal variation of soil respiration in a secondary tropical moist forest in Puerto Rico
Source: Ecol Evol. 2020 Dec 10;11(1):263–72. doi: 10.1002/ece3.7021 (PMC7790624; doi:10.1002/ece3.7021)

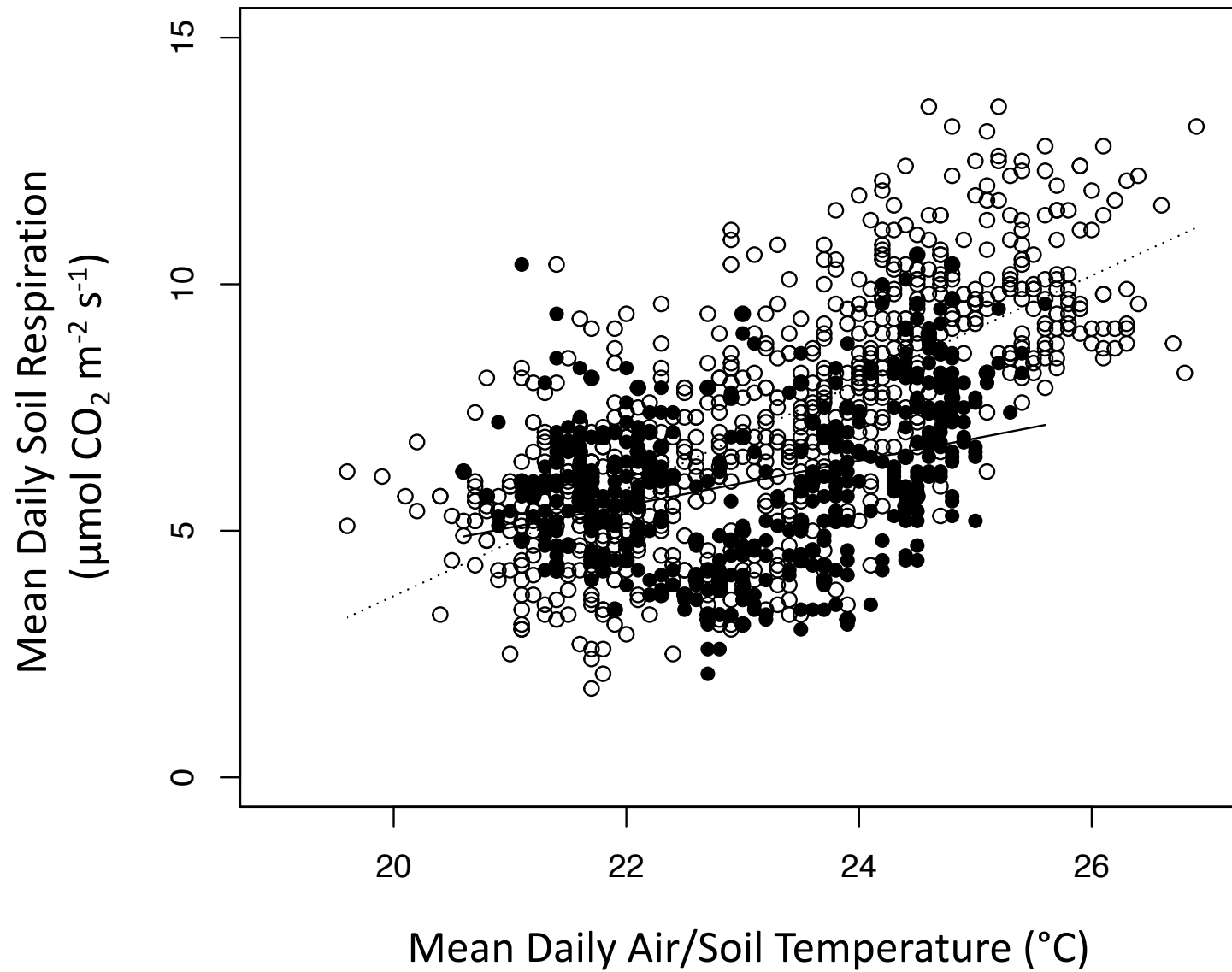

Supplement: Supplementary file 1 — Fig S1 [file ECE3-11-263-s001.pdf]

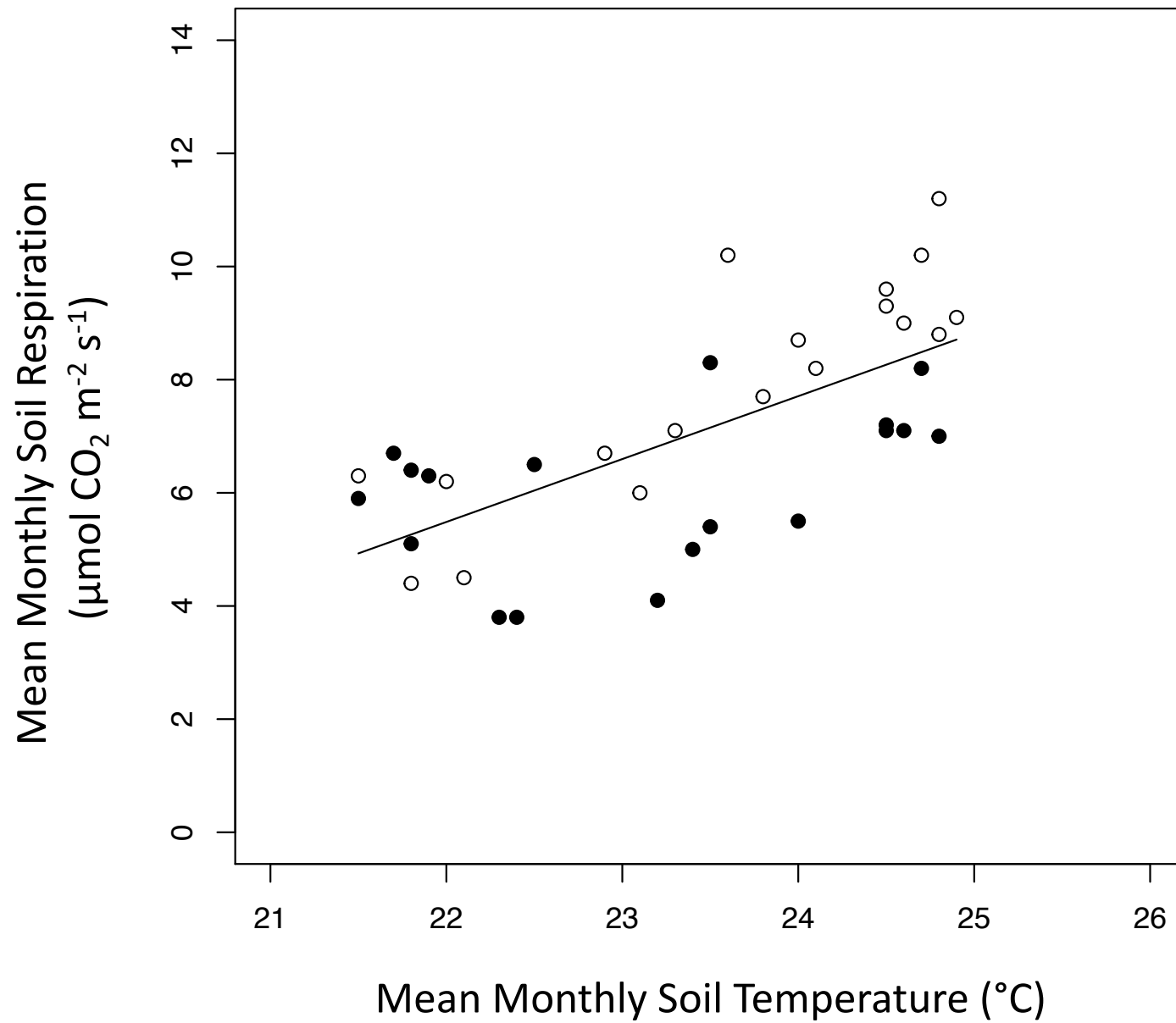

Supplement: Supplementary file 2 — Fig S2 [file ECE3-11-263-s002.pdf]

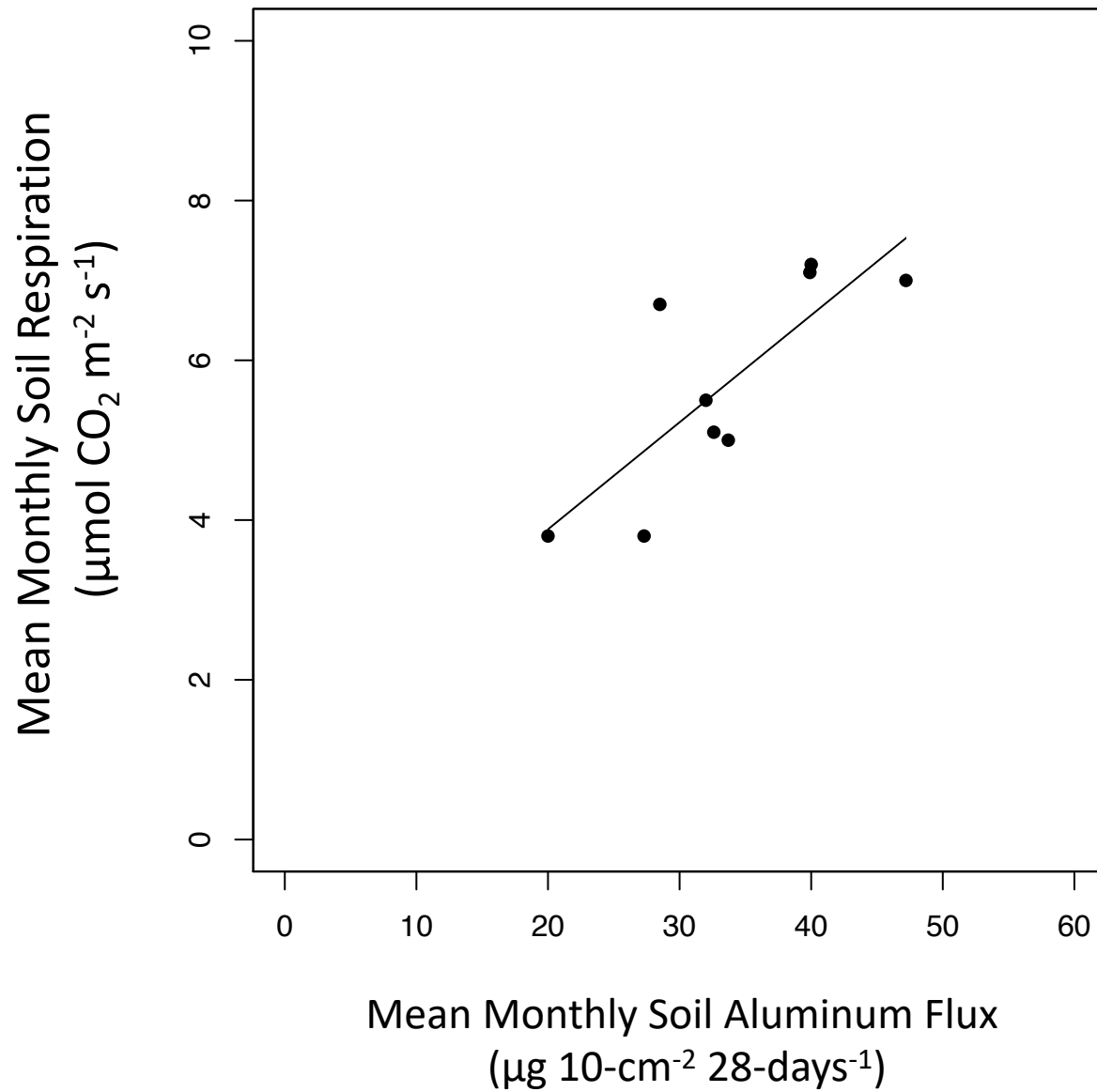

Supplement: Supplementary file 3 — Fig S3 [file ECE3-11-263-s003.pdf]
